# Transactions of the College of Physicians of Philadelphia

**Published:** 1851-07

**Authors:** 


### TRANSACTIONS OF THE COLLEGE OF PHYSICIANS OF PHILADELPHIA.

A new series of this quarterly has been commenced with a view of giving to it a more extended circulation than has heretofore been the case. We have received the first number of the new series which has been issued in excellent style by its new publishers, Messrs. Lippincott, Grambo & Co. As to the matter of these Transactions, it is not necessary that we should say anything. Among the members of the College are many of the first names in American medicine. The summary of their transactions is always valuable, and is offered to the profession at one dollar per annum, or twenty-five cents a number.

